# Effectiveness of azithromycin in aspiration pneumonia: a prospective observational study

**DOI:** 10.1186/s12879-014-0685-y

**Published:** 2014-12-10

**Authors:** Satoshi Marumo, Takashi Teranishi, Yuichi Higami, Yoshihiko Koshimo, Hirofumi Kiyokawa, Motokazu Kato

**Affiliations:** Respiratory Disease Center, Tazuke Kofukai Medical Research Institute, Kitano Hospital, 2-4-20 Ohgimachi, Kita-ku, 530-8480 Osaka, Japan; Department of Respiratory Medicine, Kishiwada City Hospital, Kishiwada, Osaka, Japan

**Keywords:** Aspiration pneumonia, Azithromycin, Nursing and healthcare-associated pneumonia

## Abstract

**Background:**

Aspiration pneumonia is an urgent health concern with high mortality and long hospitalization in industrialized and aging countries. However, there is no information about the effectiveness of azithromycin (AZM) for the treatment of aspiration pneumonia. This study investigated if AZM is effective for the treatment of aspiration pneumonia.

**Methods:**

Patients with aspiration pneumonia with no risk of multidrug-resistant pathogens were included in this prospective study at Kishiwada City Hospital from December 2011 to June 2013. Patients were divided into the ampicillin/sulbactam (ABPC/SBT) and AZM (intravenous injection) groups. The success rates of 1^st^-line antibiotic therapy, mortality, length of hospital stay, and total antibiotic costs were compared.

**Results:**

There were 81 and 36 patients in the ABPC/SBT and AZM groups, respectively. There was no significant difference in the success rate of 1^st^-line antibiotics between the groups (74.1% vs. 75.0%, respectively, *P* = 1.000). Mortality and hospitalization periods did not differ between the 2 groups (11.1% vs. 8.3%, *P* = 0.753, and 22.3 ± 7.3 days vs. 20.5 ± 8.1 days, *P* = 0.654, respectively). However, the total antibiotic costs were significantly lower in the AZM group than the ABPC/SBT group (2.19 ± 1.65 × 10,000 yen vs. 2.94 ± 1.67 × 10,000 yen, respectively, *P* = 0.034). The febrile period of the ABPC/SBT group was significantly shorter than that of the AZM group (*P* = 0.025).

**Conclusions:**

In this small prospective non-randomized observational study, we found no statistically significant differences in mortality or antibiotic failure in patients receiving AZM compared to ABPC/SBT for the treatment of patients with aspiration pneumonia who require hospital admission and have no risk of drug-resistant pathogens. Therefore, AZM may be another first choice of antibiotic treatment for patients with aspiration pneumonia when they have no risk of multidrug-resistant pathogens.

**Electronic supplementary material:**

The online version of this article (doi:10.1186/s12879-014-0685-y) contains supplementary material, which is available to authorized users.

## Background

Aspiration pneumonia is an urgent health concern with high mortality and long hospitalization periods in industrialized and aging countries [1]. In Japan’s rapidly aging society, the mortality rate of pneumonia is 1,000 times greater among people ≥85 years old than among young adults irrespective of sex; moreover, pneumonia is the leading cause of death of males ≥90 years old [2]. Most cases of pneumonia in the elderly are reported to be aspiration pneumonia [3]. Thus, aspiration pneumonia is a crucial health issue, especially in Japan.

The frequent causative pathogens of aspiration pneumonia include intraoral anaerobe-like periodontitis [4]-[6], which should be covered by empiric antibiotic therapy for aspiration pneumonia. Several guidelines state that patients with aspiration pneumonia should receive β-lactams such as ampicillin/sulbactam (ABPC/SBT) when they must be admitted to hospitals and have no risk of multidrug-resistant pathogens [2],[7].

Azithromycin (AZM) is a macrolide antibiotic used extensively for the treatment of a wide range of infections including CAP. AZM is effective not only against gram-negative aerobic bacteria, but also against anaerobic bacteria [8]. Therefore, AZM is often used to treat periodontitis [9]. However, there is no information about the effectiveness of AZM for the treatment of aspiration pneumonia. Therefore, the present study investigated if AZM is effective for the treatment of aspiration pneumonia.

## Methods

### Study design

This study was a prospective cohort study of patients with pneumonia hospitalized at Kishiwada City Hospital (a 400-bed community hospital in Kishiwada City, Osaka, Japan) from December 2011 to June 2013. Patients with pneumonia were classified as having community acquired pneumonia (CAP), hospital acquired pneumonia (HAP), or nursing and healthcare-associated pneumonia (NHCAP). According to the Japanese Respiratory Society (JRS) guideline [2], patients with aspiration pneumonia among NHCAP group B (who must be admitted to hospitals and have no risk of multidrug-resistant pathogens) were subsequently selected and categorized into the following groups on the basis of the prescribed antibiotics: ABPC/SBT, AZM (intravenous injection), and other. Treatment decisions for all study patients including type of antibiotic therapy administered were not standardized and were made by physicians. We compared the success rates of 1^st^-line antibiotic therapy, mortality, length of hospital stay, and total antibiotic costs among groups. This study was approved by the ethics committee of Kishiwada City Hospital and all participants provide written informed consent prior to participation on admission.

### Data collection

All data were collected prospectively. The following data were recorded on admission: age, sex, sociodemographics, Eastern Cooperative Oncology Group (ECOG) Performance Status, medical treatments, physical examination findings, laboratory parameters, and chest radiograph findings. CURB-65 and A-DROP were calculated on admission to evaluate the severity of pneumonia [2],[10]. CURB-65 consisted of the following 5 score: 1) confusion of new onset, 2) blood urea nitrogen (BUN) >19 mg/dL, 3) respiratory rate ≥30/min, 4) systolic blood pressure <90 mmHg or diastolic blood pressure <60 mmHg, and 5) age ≥65 years. A-DROP was a severity score modified from CURB-65 to adjust Japanese healthcare system [11]. A-DROP consisted of the following 5 score: 1) age (men ≥70 years, women ≥75 years), 2) dehydration (BUN ≥21 mg/dL), 3) respiratory failure (SpO2 ≤ 90% or PaO2 ≤ 60 Torr), 4) orientation disturbance, and 5) systolic blood pressure ≤90 mmHg [12]. The clinical outcomes were the success rate of the 1^st^-line antibiotic therapy, in-hospital mortality, length of hospital stay, and total antibiotic costs. Febrile periods were also compared between groups.

### Microbiological studies

Microbiological diagnosis was based on Gram stain and culture samples (i.e., sputum, pleural fluid, or blood), rapid urinary antigen test for *Streptococcus pneumoniae* (BinaxNOW, Scarborough, MA, USA), and serum *Mycoplasma pneumoniae*-specific IgM antibody. Gram staining was performed by trained laboratory medical technologists, and culture results were recorded semi-quantitatively. An etiological diagnosis was established if any of the following criteria were met: (1) moderate to heavy growth from sputum samples suitable for evaluation (i.e., the presence of >10 polymorphonuclear leukocytes and <10 squamous cells per low-power field) with compatible Gram stain; (2) positive culture in pleural fluid; (3) positive blood culture if no other source was identified; (4) positive urinary antigen test for *S. pneumoniae*; (5) positive rapid influenza diagnostic test and other microbial etiology was negative.

### Definitions

Pneumonia was defined as a new infiltrate together with signs and symptoms of lower respiratory tract infection. HAP was defined as pneumonia that occurred ≥48 h after acute hospital admission. NHCAP was defined according to the JRS guidelines [2] and included patients who met at least one of the following criteria: (1) residence in a long-term care hospital or a nursing home; (2) discharge from a hospital in the past 90 days; (3) elderly or disabled person requiring care (i.e., performance status 3 or 4); and (4) an outpatient who regularly receives infusion therapy (i.e., chronic dialysis, antibiotics, cancer chemotherapy, and immunosuppressive drugs). NHCAP group B met both of the following criteria: (1) requirement of hospitalization and (2) no risk factors for multidrug-resistant pathogens (i.e., no antibiotics within the preceding 90 days, current tube feeding, or history of methicillin-resistant *Staphylococcus aureus* [MRSA] isolation). Patients were classified as CAP if they did not meet the criteria for HAP or NHCAP. According to the NHCAP guideline [2], aspiration pneumonia was defined as being pneumonia that develops in patients in whom dysphagia and aspiration is known to occur (or is strongly suspected). The diagnosis of aspiration pneumonia was categorized into three groups; definitive cases (direct observation of aspiration by videofluoroscopic (VF) examination of swallowing), probable cases (presence of functional dysphagia examined by bedside assessment of swallowing function, arterial oxygen saturation monitoring during swallowing, repetitive saliva swallowing test, water swallowing test, simple swallowing provocation test), and possible cases (presence of risk factors for oropharyngeal aspiration but without direct observation of aspiration nor functional dysphagia). Swallowing function tests (VF and bedside examinations) were conducted when the pneumonia of the patient had been improved and started their meal according to the physician’s decision (not to the documented criteria). Patients were excluded if they were considered to have other diseases that distinguished them from pneumonia at follow-up. Patients were also excluded if their etiological diagnosis was influenza.

The clinical response to antibiotic treatment was determined by assessing signs and symptoms of respiratory infections as well as comparing the baseline and end-of-treatment chest X-rays. Clinical efficacy was assessed mainly based on the evaluation of body temperature, WBC count, CRP, chest X-ray findings, signs and symptoms of pneumonia generally according to the criteria for the evaluation of effectiveness in clinical efficacy recommended by Japanese Respiratory Society [2]. Total antibiotic costs were calculated, including all intravenous and oral antibiotics administered from admission to discharge from our hospital.

MRSA, *Pseudomonas aeruginosa*, *Acinetobacter* species, and extended-spectrum β-lactamase-producing *Enterobacteriaceae* were considered as multidrug-resistant pathogens on the basis of the 2005 IDSA/ATS guidelines.

### Statistical analysis

Comparisons between groups were made using the *χ*^2^ test or Fisher’s exact test for categorical variables and Student’s *t*-test, and the Mann–Whitney *U*-test for continuous variables. Logistic regressions were performed to investigate the clinical outcomes after adjustment for the severity assessed by CURB-65 and ADROP. Febrile periods were analyzed by the Kaplan–Meier method and compared by the log-rank test. The level of statistical significance was set at *P* < 0.05. All data were processed and analyzed using SPSS version 20.0 (SPSS, Chicago, IL, USA)

## Results

### Patient characteristics

**Figure 1:**
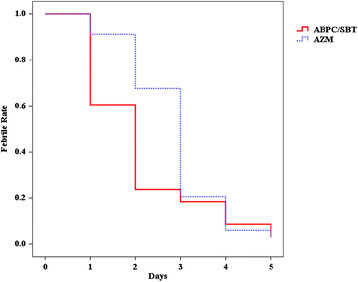
**Kaplan–Meier Curve of Febrile Periods.** The febrile period of the ABPC/SBT group tended to be shorter than that of the AZM group. ABPC/SBT: ampicillin/sulbactam; AZM, azithromycin.

During the study period, 498 patients were admitted to our hospital. The Kaplan–Meier plot of febrility is shown in Figure 1. Briefly, 192 patients were diagnosed as NHCAP group B, including 148 patients with aspiration pneumonia. Then, 31 patients were excluded because they were treated with other antibiotics. Finally, 81 and 36 patients were included in the ABPC/SBT and AZM groups, respectively. The baseline characteristics of the subjects are shown in Table 1. There were no significant differences between the AZM and ABPC/SBT groups with respect to sex, age, severity, and diagnostic category of aspiration pneumonia.

**Table 1:** **Baseline subject characteristics**

		ABPC/SBT ( *n* = 81)	AZM ( *n* = 36)	*P* value
Sex	female/male	23/58	12/24	0.663
Age		78.9 ± 8.57	78.6 ± 9.15	0.874
Severity	CURB-65	1.75 ± 0.80	1.69 ± 0.79	0.712
	A-DROP	1.57 ± 0.91	1.50 ± 0.94	0.717
Diagnosis of Aspiration Pneumonia	Definitive	8 (9.9%)	2 (5.6%)	0.722
	Probable	69 (85.2%)	30 (83.3%)	0.787
	Possible	4 (4.9%)	4 (11.1%)	0.248
Pathogen	*S. pneumoniae*	17 (21.0%)	9 (25.0%)	0.637
	*H. influenzae*	5 (6.2%)	2 (5.6%)	1.000
	*E. coli*	2 (2.5%)	0 (0.0%)	1.000
	*P. aeruginosa*	1 (1.2%)	1 (2.8%)	0.523
	MSSA	9 (11.1%)	3 (8.3%)	0.753
	MRSA	1 (1.2%)	0 (0.0%)	1.000
	unknown	44 (52.3%)	18 (50.0%)	0.692

Analyzed by Fisher’s exact test or Mann–Whitney *U*-test.

ABPC/SBT, ampicillin/sulbactam.

AZM, azithromycin.

MSSA, methicillin-sensitive *Staphylococcus aureus.*

MRSA, methicillin-resistant *Staphylococcus aureus.*

### Pathogen distribution

Blood and sputum cultures were performed in 88 (75.2%) and 98 (89.7%) patients, respectively. Urinary antigen tests were performed in 43 patients (36.8%). An etiological diagnosis was established in 55 patients (47.0%); among them, 43 (36.8%), 9 (7.7%), 5 (5.1%), and 1 (0.85%) were diagnosed on the basis of sputum cultures, urinary antigen tests, blood culture, and pleural effusion, respectively. There was no significant difference in the frequency of an etiological diagnosis between the ABPC/SBT and AZM groups (45.7% vs. 50.0%, respectively, *P* = 0.692).

*S. pneumoniae* was the most frequent causative pathogen in both the ABPC/SBT and AZM groups, accounting for 21.0% and 25.0%, respectively (Table 1). All isolated pneumococcal strains were penicillin sensitive (minimum inhibitory concentration [MIC] ≤ 2 μg/mL), according to the Clinical and Laboratory Standards Institute (CLSI) cut-offs [13]. The second most frequent causative pathogen in both groups was methicillin-sensitive *Staphylococcus aureus*. There was no significant difference between the isolation rates of MRSA or *P. aeruginosa* between groups.

### Clinical outcomes

The clinical outcomes of the 2 groups are shown in Table 2. There was no significant difference in the success rate of 1^st^-line antibiotics between the ABPC/SBT and AZM groups (74.1% vs. 75.0%, respectively, *P* = 1.000). Mortality and hospitalization periods did not differ significantly between groups (11.1% vs. 8.3%, *P* = 0.753; 22.3 ± 7.3 vs. 20.5 ± 8.1 days, *P* = 0.654, respectively). However, the total antibiotic costs were significantly lower in the AZM group than the ABPC/SBT group (2.19 ± 1.65 × 10,000 yen vs. 2.94 ± 1.67 × 10,000 yen, respectively, *P* = 0.034). There are two patients with positive serum *Mycoplasma pneumoniae* IgM antibody in the AZM group. On the other hand, there was no patient in the ABPC/SBT group. Excluding the two patients in the AZM group, we compared the clinical outcome again. There was no significant difference in the success rate of 1st-line antibiotics between the ABPC/SBT and AZM groups (74.1% vs. 74.3%, respectively, P = 1.000). Mortality and hospitalization periods did not differ significantly between groups (11.1% vs. 8.8%, P = 1.00; 22.3 ± 7.3 vs. 20.7 ± 8.0 days, P = 0.674, respectively). The total antibiotic costs were significantly lower in the AZM group than the ABPC/SBT group (2.13 ± 1.62 × 10,000 yen vs. 2.94 ± 1.67 × 10,000 yen, respectively, P = 0.024).

**Table 2:** **Clinical outcomes**

	ABPC/SBT ( *n* = 81)	AZM ( *n* = 36)	*P* value
Success rate of 1^st^-line antibiotics	60 (74.1%)	24 (75.0%)	1.000
Mortality	9 (11.1%)	3 (8.3%)	0.753
Hospitalization period (days)	22.3 ± 7.3	20.5 ± 8.1	0.654
Total antibiotic costs (×10,000 yen)	2.94 ± 1.67	2.19 ± 1.65	0.034

Analyzed by Fisher’s exact test or Mann–Whitney *U*-test.

ABPC/SBT, ampicillin/sulbactam.

AZM, azithromycin.

The adjusted odds ratios (OR) of the success rate of 1^st^-line antibiotics and mortality were calculated by logistic regressions. The OR for the success rate of 1^st^-line antibiotics comparing ABPC/SBT group to AZM group were 0.901 (95% confidential interval (CI): 0.360 – 2.258) and 0.887 (95% CI: 0.352 – 2.234) after adjustment by CURB-65 and ADROP, respectively. The OR for mortality comparing ABPC/SBT group to AZM group were 1.297 (95% CI: 0.310 – 5.433) and 1.258 (95% CI: 0.293 – 5.408) after adjustment by CURB-65 and ADROP, respectively.

Patients received ABPC/SBT (2:1) 1.5 g intravenously every 8 h for 7–14 days. For patients with decreased estimated glomerular filtration rate (eGFR), the q8h dosage of ABPC/SBT was adjusted as follows: patients with an eGFR 15–29 and <14 mL/min were administered 1.5 g every 12 and 24 h, respectively. Patients received AZM 500 mg intravenously every 24 h for 5 days, followed by switching to AZM 500 mg orally every 24 h for 3 days. In patients successfully treated with 1^st^-line antibiotics, ABPC/SBT was administered for an average of 9.03 ± 1.80 days. In 57 of the 60 successful cases, ABPC/SBT was continued until the end of treatment without switching to oral antibiotics. On the other hand, AZM was administered for 5 days in all successfully treated cases. In 11 of 24 successful cases, intravenous AZM was switched to oral AZM (500 mg/day for 3 days).

The Kaplan–Meier curves of the febrile periods are shown in Figure 1. The log-rank test showed the febrile period of the ABPC/SBT group was significantly shorter than that of the AZM group (*P* = 0.025).

## Discussion

The present study is the first to demonstrate the noninferiority of AZM to ABPC/SBT for the treatment of patients with aspiration pneumonia who need to be admitted to hospitals and have no risk of multidrug-resistant pathogens (i.e., NHCAP group B). The success rates of 1^st^-line antibiotic therapy, mortality rates, and hospitalization duration were similar between patients treated with AZM and ABPC/SBT. However, the total antibiotic costs were significantly lower in patients treated with AZM than ABPC/SBT. The febrile period of the ABPC/SBT group was significantly shorter than that of the AZM group.

Pneumonia has traditionally been classified as either community or hospital acquired (CAP and HAP, respectively) depending whether it developed in outpatient or inpatient settings. The joint guidelines proposed by the American Thoracic Society (ATS) and the Infectious Diseases Society of America (IDSA) define a new category of pneumonia: healthcare-associated pneumonia (HCAP) [7]. Many subsequent studies from various countries revealed that HCAP is a clinically heterogeneous disease and that the populations of patients with HCAP vary among countries depending on the healthcare environment including social health insurance systems. In order to adjust to Japan’s healthcare system, HCAP was changed to “nursing and healthcare-associated pneumonia” (NHCAP) in 2011 by the Japanese Respiratory Society (JRS) [2]. Large proportions of NHCAP and HCAP cases are aspiration pneumonia [3].

The pathogens of aspiration pneumonia are reported to include not only common bacteria of CAP such as *S. pneumoniae*, but also intraoral anaerobic bacteria such as *Fusobacterium*, *Peptostreptococcus*, *Bacteroides*, and *Prevotella* [4]-[6]. Therefore, HCAP and NHCAP guidelines recommend administering penicillin with β-lactamase inhibitor (ABPC/SBT or piperacillin/tazobactam) or carbapenems (imipenem/cilastatin or meropenem) [2],[7]. Because these β-lactams must be administered 3 times or more per day according to pharmacokinetic/pharmacodynamic theory [14], patients with aspiration pneumonia require hospitalization. On the other hand, AZM only requires once daily administration. Therefore, the present study suggests outpatient parenteral antimicrobial therapy (OPAT) could be administered to patients with aspiration pneumonia if their dysphagia is mild such that they are able eat a normal diet. OPAT for patients with aspiration pneumonia might provide several benefits not only to the patients themselves, but to society as well [15]. OPAT could reduce the costs of healthcare systems as well as hospitalization-induced psychological stress in patients. Nevertheless, an interventional study is required to test these hypotheses.

In the present study, Kaplan–Meier analysis revealed that the ABPC/SBT group had a significantly shorter febrile period than that of the AZM group. This may be partly due to the different antimicrobial activities of ABPC/SBT and AZM: ABPC/SBT has bactericidal activity, whereas AZM has bacteriostatic activity [16]-[18]. Therefore, ABPC/SBT rapidly ameliorates the inflammation of bacterial pneumonia by killing pathological bacteria. However, this does not mean that ABPC/SBT is more effective than AZM in cases of severe pneumonia. Combination therapy is administered for severe pneumonia, in which macrolide-containing combinations improve mortality rate [19]. Moreover, these effects have been demonstrated even in cases involving pneumonia caused by macrolide-resistant pathogens. Several studies indicate that the anti-inflammatory effects of macrolide could account for the improved mortality rate.

As mentioned above, total antibiotic costs were significantly lower in the AZM group than the ABPC/SBT group. In the AZM group, AZM 500 mg was administered intravenously for 5 days before switching to oral administration of the same dose for 3 days. On the other hand, in the ABPC/SBT group, intravenous administration of ABPC/SBT was required to continue until the end of antibiotic therapy. This is because the approved dose for the oral administration of penicillin with a β-lactamase inhibitor is insufficient for the treatment of pneumonia in Japan. Therefore, ABPC/SBT was administered intravenously until the end of treatment in the present study. Thus, the present results demonstrate AZM is comparatively cost effective. However, randomized controlled studies are required to confirm this finding.

Recent reports demonstrate *S. pneumoniae* has high resistance to macrolides worldwide. In Japan, clarithromycin-resistant strains are reported to make up over 50% of all of *S. pneumoniae* strains [20]. Furthermore, the proportion of AZM-resistant strains is reported to be as high as that of clarithromycin-resistant strains. In the present study, the resistance rate of isolated *S. pneumoniae* to clarithromycin was as high as 68.0% (17 out of 25). However, the success rate of intravenous AZM was acceptably high. Thus, there is a discrepancy between the efficacy of clarithromycin in vitro and the effectiveness of intravenous AZM in vivo. A previous study reports similar results, i.e., intravenous-to-oral azithromycin therapy demonstrated excellent clinical and bacteriological effects on moderate-to-severe pneumococcal pneumonia despite a high MIC and resistance gene development [21]. This discrepancy can be explained by the unique properties of AZM, including phagocyte delivery, extremely long half-life, inhibitory effect on *S. pneumoniae* pneumolysin production, and anti-inflammatory and immunomodulatory activities [22]-[25].

This study has several limitations that should be mentioned. First, the present study was not a randomized controlled study but an observational study. Many confounders may lie hidden. However, the administration of either AZM or ABPC/SBT was comparable and the selection bias of antibiotics (i.e., AZM vs. ABPC/SBT) may be small, because the baseline characteristics were very similar between the two groups, including the severity of pneumonia and pathogens. Second, no discharge or antibiotic withdrawal criteria were defined, which might have influenced hospitalization duration and total antibiotic costs. Third, anaerobic cultures for sputum sample were not performed in the present study, though anaerobic bacteria are regarded as important causative pathogens for aspiration pneumonia. Forth, serum antibodies for *Chlamydophila* were not measured in the present study, though potent activities of AZM against atypical pathogens including *Chlamydophila* might have influenced the clinical outcomes.

AZM is appropriate for aspiration pneumonia for the following reasons. First, AZM could cover most causative pathogens of aspiration pneumonia from gram-positive cocci such as *S. pneumoniae* to anaerobic bacteria, which are common pathogens of periodontitis. Second, OPAT could be used to treat aspiration pneumonia by selecting intravenous antibiotics with AZM. Third, the unique effects of AZM may play beneficial roles in patients with aspiration pneumonia [25].

## Conclusions

In this small prospective non-randomized observational study, we found no statistically significant differences in mortality or antibiotic failure in patients receiving AZM compared to ABPC/SBT. Therefore, AZM may be another first choice of antibiotic treatment for patients with aspiration pneumonia who require hospitalization and have no risk of multi-drug resistant pathogens.

## Authors’ original submitted files for images

Below are the links to the authors’ original submitted files for images. Authors’ original file for figure 1
