# Supplementary figures and images for: Effectiveness of azithromycin in aspiration pneumonia: a prospective observational study
Source: BMC Infect Dis. 2014 Dec 10;14:685. doi: 10.1186/s12879-014-0685-y (PMC4265472; doi:10.1186/s12879-014-0685-y)

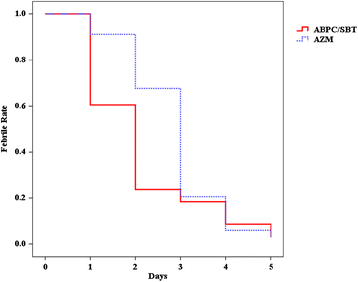

Supplement: Supplementary file 1 — Authors’ original file for figure 1 [file 12879_2014_685_MOESM1_ESM.gif]
